# Hidden gradients before form: a spatial transcriptomic atlas of wheat spike patterning

**DOI:** 10.1093/plcell/koaf286

**Published:** 2025-12-18

**Authors:** Min-Yao Jhu, Travis A Lee

**Affiliations:** Assistant Features Editor, the Plant Cell, American Society of Plant Biologists; Crop Science Centre, Department of Plant Sciences, University of Cambridge, Cambridge, United Kingdom; Plant Biology Laboratory, Salk Institute for Biological Studies, La Jolla, CA, United States

Few sights are as emblematic of agriculture as the wheat spike—an elegant, lanceolate-shaped inflorescence that determines grain yield. The highly productive wheat spike relies on precise developmental timing; however, its architecture harbors a paradox: basal spikelets, although initiated first, are often delayed in development and remain rudimentary, failing to produce grain (Backhaus et al. 2022, 2023). The cause of this developmental imbalance has remained an open question, and understanding the gene expression patterns coordinating this crucial apical–basal axis has long been hindered by bulk tissue transcriptome approaches, obscuring regional and cellular variation.

Here, Katie A. Long, Ashleigh Lister, and colleagues **(Long et al. 2025)** successfully adapt multiplexed error robust fluorescence in situ hybridization (MERFISH), an imaging-based spatial transcriptomics technique (Chen et al. 2015; Moffitt et al. 2016), to the anatomically complex wheat inflorescence, providing an unprecedented, cellular-resolution view of gene activity along spikelet development. Their developmental survey spans 4 key developmental stages, including (1) the Late Double Ridge (W2.5) stage, when leaf and spikelet ridges are first visible; (2) the Lemma Primordia (W3.25), marking the initiation of glumes and lemmas; (3) the Terminal Spikelet (W4), when the uppermost spikelet forms and floral organs begin differentiating; and (4) Carpel Extension (W5), corresponding to carpel growth around the ovule. By localizing transcripts in situ for 200 developmental genes across more than 50,000 cells, the authors identified 18 distinct expression domains (EDs) and their marker genes, constructing a high-resolution map of spatiotemporal gene organization during spikelet and floral development that elucidates transcriptional signatures associated with cell type, developmental stage, and the intersection therein (Fig. 1a-c).

**Figure 1.**
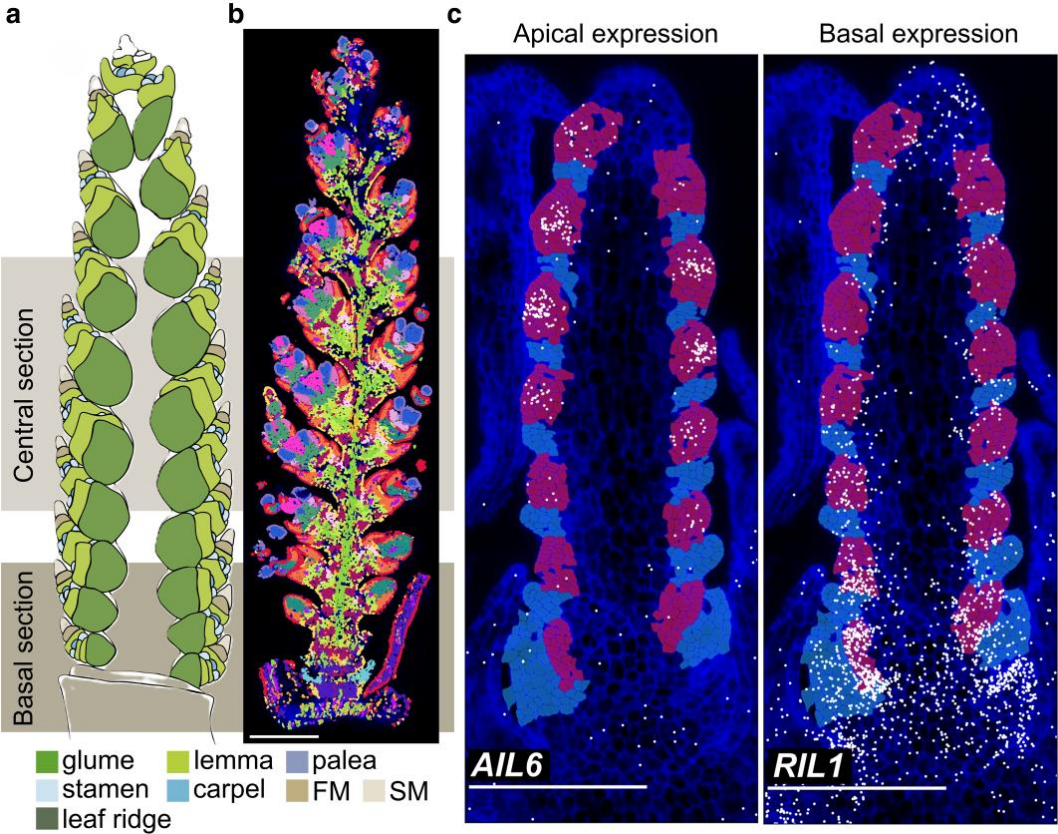
Spatial transcriptomics reveals distinct gene expression domains and developmental gradients in wheat spikes. a) Diagram of wheat inflorescence stages, showing the W5 (carpel-extending) stage. b) Cellular-resolution map of transcriptional domains identified by Leiden clustering at W5. Scale bar = 100 µm. c) Spatial gene expression patterns illustrating early developmental gradients at the Double Ridge stage (W2.5). AIL6 and RIL1 show contrasting apical–basal expression profiles. LR cells are highlighted in blue and AM cells in pink. Adapted from Long et al. (2025), Supplementary Figure 1D and Figures 2C and 5F, G.

Spatial clustering and domain-enrichment analysis allow these EDs to be mapped onto recognizable tissues and floral organs. In mature spikes (W5), 8 floral and bract-related domains emerge (Fig. 1b), corresponding to glume/lemma, palea, lodicule, stamen, and carpel identities. Within these regions, MERFISH resolves combinatorial expression of MADS-box transcription factors consistent with the grass ABCDE model—for example, B-class genes in lodicules and stamens, C-class genes in stamens and carpels, and E-class factors in partially overlapping patterns. These spatially defined coexpression signatures validate the approach and refine how floral identity programs are organized across the developing wheat floret.

A central finding is the early transcriptional asymmetry along the apical–basal axis, which emerges before visible spikelet formation at the W2.5. At this early stage, the developing spike initiates alternating axillary meristems (AMs) and leaf ridges (LRs). Spatial transcriptomic analysis revealed that central AMs differ markedly from basal AMs in their gene expression profiles. In central AMs, expression of genes linked to meristem maintenance and floral transition was enriched and strongly expressed, including genes such as the ortholog to *AINTEGUMENTA-LIKE6* (*AIL6*), which is associated with floral meristem identity. In contrast, basal AMs and neighboring LRs show higher expression of genes associated with indeterminate branch meristems (BM) in rice, including the ortholog to *RACHIS-LIKE 1* (*RIL1*) (Fig. 1c). These distinct molecular signatures indicate that only central AMs acquire early spikelet meristem (SM) competency, providing a clear transcriptional basis for the developmental delay observed in basal spikelets.

By integrating cellular resolution with spatial context across multiple developmental stages, Long et al. provide new insights into the genetic programs that pattern the wheat spike and highlight key regulators that contribute to early axial patterning. To maximize community access to the dataset, all raw and processed data, including domain- and cell-level maps developed by the authors, are publicly available through an interactive WebAtlas interface (www.wheat-spatial.com) (Long et al. 2025). This work establishes a robust framework for high-resolution spatial transcriptomics in complex plant organs. Beyond wheat, this work demonstrates the transformative potential of spatial transcriptomics for plant developmental biology, revealing not only where genes are expressed but also when and how those patterns interact to create form.

## Recent related articles in *The Plant Cell*:

Liu et al. (2025) uncovered 2 opposing transcriptional modules that fine-tune *VRT-A2* expression in wheat spikes—an AP2/ERF repressor (TaMFS1) and an activator (TaSSRP1)—showing that modulating this regulatory balance enhances grain length and weight without reducing spikelet fertility.
Li et al. (2021) demonstrated that interactions between SQUAMOSA- and SHORT VEGETATIVE PHASE–clade MADS-box proteins coordinate wheat meristem transitions, showing that *SQUAMOSA* genes repress *VRT2/SVP* activity to promote spikelet identity and floral development.
Adamski et al. (2021) showed that an intron-1 *cis*-regulatory variant driving ectopic, dosage-dependent *VRT-A2* expression causes elongated glumes, lemmas, and grains in wheat.

## Data Availability

NA.

## Dive Curated Terms

The following phenotypic, genotypic, and functional terms are of significance to the work described in this paper:

AIL6 Gramene: AT5G10510
AIL6 Araport: AT5G10510
